# Supplementary material for: BA.1/BA.5 Immunogenicity, Reactogenicity, and Disease Activity after COVID-19 Vaccination in Patients with ANCA-Associated Vasculitis: A Prospective Observational Cohort Study
Source: Viruses. 2023 Aug 21;15(8):1778. doi: 10.3390/v15081778 (PMC10458303; doi:10.3390/v15081778)
Supplement: Supplementary file 1 [file viruses-15-01778-s001.zip › viruses-2520543-supplementary.pdf]

viruses

MDPI

Supplementary Materials

BA.1/BA.5 Immunogenicity, Reactogenicity, and Disease Activity after COVID-19 Vaccination in Patients with ANCA-Associated Vasculitis: A Prospective Observational Cohort Study

Table S1. Cutoff values of the SARS-CoV-2 specific bead-based multiplex assay.

Target	Cutoff (MFI)
SARS-CoV-2 Full Spike	6800
SARS-CoV-2 Spike S1	2700
SARS-CoV-2 Spike RBD	5800
SARS-CoV-2 Spike S2	3200
SARS-CoV-2 Nucleocapsid Protein	5900

SARS, severe acute respiratory syndrome; MFI, mean fluorescence intensity.

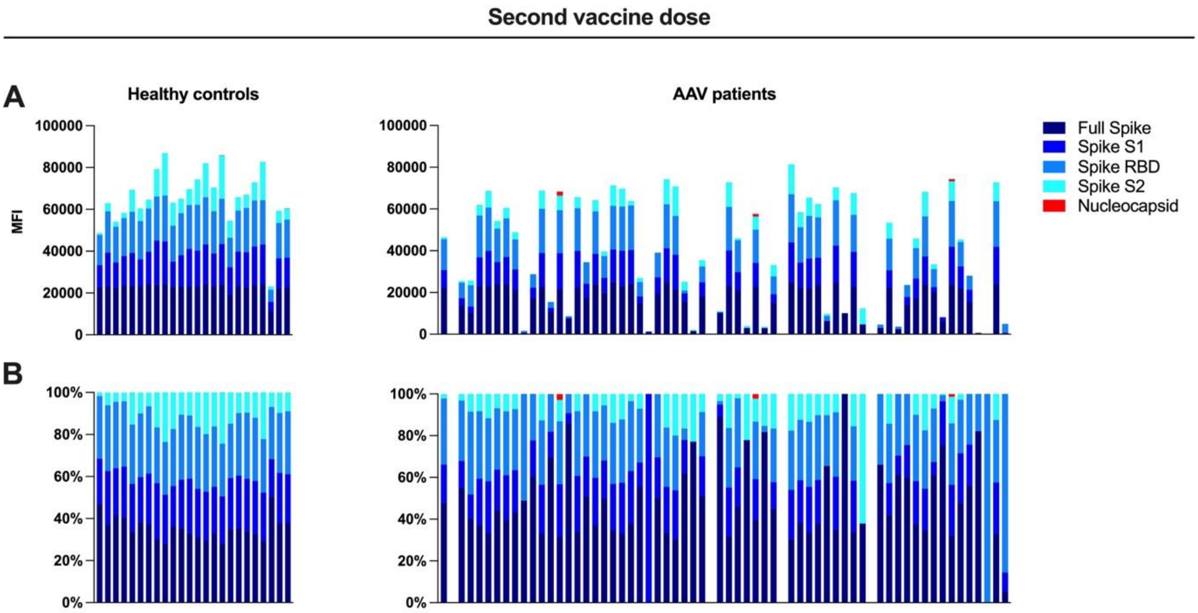

Figure S1. Antibodies against different SARS-CoV-2 target epitopes in ANCA-associated vasculitis patients and healthy controls after the second vaccine dose. Multiplex analysis of antibodies against the full spike, the S1 spike, the receptor-binding domain (RBD) of the spike, the S2 spike protein, and the nucleoprotein of SARS-CoV-2 measured in 64 ANCA-associated vasculitis patients and 24 healthy controls after a second vaccine dose matched for type of vaccine, age, and sex. (**A**) The mean fluorescence intensity (MFI) value of the reactivity in ANCA-associated vasculitis patients and healthy controls is represented on the y-axis. The x-axis represents the sample number. (**B**) Proportional contribution (%) of each antibody target to the total MFI value for ANCA-associated vasculitis patients and healthy controls, respectively.

Side effects questionnaire

1. **Side effects after 1st vaccination:**

◯ yes ◯ no

2. **If yes, please mark the appropriate side effects you had:**

◯ local events (such as pain at injection site, redness, swelling)
◯ fever ◯ fatigue ◯ headache ◯ chills
◯ vomiting ◯ diarrhea ◯ muscle pain ◯ joint pain
◯ swollen lymph nodes ◯ others, such as ___________________

3. **In response to above mentioned side-effects, I took the following medication**

◯ NSAID (i.e. Ibuprofen)
◯ Paracetamol
◯ Metamizol (i.e. Novalgin)
◯ Aspirin
◯ others, such as ___________________

1. **Side effects after 2nd vaccination:**

◯ yes ◯ no

2. **If yes, please mark the appropriate side effects you had:**

◯ local events (such as pain at injection site, redness, swelling)
◯ fever ◯ fatigue ◯ headache ◯ chills
◯ vomiting ◯ diarrhea ◯ muscle pain ◯ joint pain
◯ swollen lymph nodes ◯ others, such as ___________________

3. **In response to above mentioned side-effects, I took the following medication**

◯ NSAID (i.e. Ibuprofen)
◯ Paracetamol
◯ Metamizol (i.e. Novalgin)
◯ Aspirin
◯ others, such as ___________________

1. **Side effects after 3nd vaccination:**

◯ yes ◯ no

2. **If yes, please mark the appropriate side effects you had:**

◯ local events (such as pain at injection site, redness, swelling)
◯ fever ◯ fatigue ◯ headache ◯ chills
◯ vomiting ◯ diarrhea ◯ muscle pain ◯ joint pain
◯ swollen lymph nodes ◯ others, such as ___________________

3. **In response to above mentioned side-effects, I took the following medication**

◯ NSAID (i.e. Ibuprofen)
◯ Paracetamol
◯ Metamizol (i.e. Novalgin)
◯ Aspirin
◯ others, such as ___________________
